# Characterization of Novel Src Family Kinase Inhibitors to Attenuate Microgliosis

**DOI:** 10.1371/journal.pone.0132604

**Published:** 2015-07-10

**Authors:** Gunjan D. Manocha, Kendra L. Puig, Susan A. Austin, Kathleen Seyb, Marcie A. Glicksman, Colin K. Combs

**Affiliations:** 1 Department of Basic Sciences, University of North Dakota, School of Medicine and Health Sciences, Grand Forks, ND, 58203, United States of America; 2 Department of Anesthesiology, Mayo Clinic, Rochester, MN, 55905, United States of America; 3 Laboratory for Drug Discovery in Neurodegeneration, Harvard NeuroDiscovery Center, Brigham and Women's Hospital, Cambridge, MA, 02139, United States of America; Indiana School of Medicine, UNITED STATES

## Abstract

Microgliosis is a major hallmark of Alzheimer’s disease (AD) brain pathology. Aβ peptide is hypothesized to act as a stimulus for microglia leading to activation of non-receptor tyrosine kinases and subsequent secretion of pro-inflammatory cytokines. Therefore, the signaling pathways mediating microglial activation may be important therapeutic targets of anti-inflammatory therapy for AD. Four novel compounds were chosen after high throughput screening kinase activity assays determined them as potential Lyn kinase inhibitors. Their kinase inhibitory and anti-inflammatory effect on Aβ-stimulated activation was assessed using the murine microglial cell line, BV2. Cells were treated with the compounds to determine effects on active, phosphorylated levels of Src family kinases, Src and Lyn, as well as MAP kinases ERK, JNK and p38. Only one compound, LDDN-0003499, produced a dose dependent decrease in basal levels of active, phosphorylated Src and Lyn in the BV2 cells. LDDN-0003499 treatment also attenuated the Aβ-stimulated increase in active, phosphorylated levels of Lyn/Src and TNFα and IL-6 secretion. This study identifies a novel small molecule Src family tyrosine kinase inhibitor with anti-inflammatory effects in response to Aβ stimulation of microglia. Further *in vitro*/*in vivo* characterization of LDDN-0003499 as well as structural modification may provide a new tool for attenuating microglial-mediated brain inflammatory conditions such as that occurring in AD.

## Introduction

Microglia are the resident macrophages of the brain comprising 5–20% of all the cells in the brain [1, 2]. In response to a number of pathological states including brain injury, ischemia, infection and neurodegenerative diseases, resting microglia may become activated. The activated microglia, also called “reactive microglia”, may present with not only an altered morphologic phenotype but also altered secretion of pro-inflammatory cytokines such as IL-6, IL-1β and TNF-α [3–7]. Data suggests that chronic microglial activation contributes to development and progression of a number of neurodegenerative diseases [8–12]. Therefore, strategies aimed at limiting initial or maintained microglial activation during disease are high priority areas for attenuating the inflammatory component of select brain pathophysiology.

For example, Alzheimer’s disease (AD) brains are characterized by the presence of abundant reactive microglia [13–16]. A large degree of both *in vitro* and *in vivo* data has established a strong association between microglia-mediated inflammation and AD [17–21]. Microglia are associated with Aβ containing plaques and Aβ is well known to be a potent, proinflammatory activator of microglia [22–24]. Therefore, modulating microglial phenotype to prevent pro-inflammatory changes in the brain may be useful therapeutically in preventing or reducing AD pathology [6, 7, 9, 25–29].

Tyrosine kinase-mediated signaling pathways are characteristically involved in the activation response of microglia to stimulation. Compared to other neural cell types, protein phosphotyrosine levels appear elevated both *in vitro* and *in vivo* in microglia [30]. In fact, Aβ plaque associated microglia demonstrate increased phosphotyrosine immunoreactivity in AD brains compared to controls suggesting an active tyrosine kinase-mediated signaling response is occurring in diseased brain cells [31, 32]. To determine whether Aβ interaction may be responsible for specific tyrosine kinase-dependent changes in microglial phenotype we, as well as others, have demonstrated using human monocytic lineage cells [24, 33–35], murine microglia cultures [36–38] and intracerebroventricular infusion [39, 40] that Aβ fibrils and oligomers stimulate increased active levels of multiple non-receptor tyrosine kinases in microglia that are required for acquisition of a proinflammatory phenotype. In particular, members of the Src family of kinases including Src and Lyn appear activated by Aβ stimulation [39, 40]. This suggests that this family of kinases, particularly Lyn due to its enrichment in immune cells [41–43], may be attractive targets for novel anti-inflammatory drug development in AD.

In this study, we characterize the ability of four novel Src family kinase inhibitors to attenuate microgliosis *in vitro*. One particular compound, LDDN-0003499, was able to attenuate basal levels of active, phosphorylated Lyn and Src but not ERK, JNK, or p38 kinases in the BV2 microglial cell line. LDDN-0003499 treatment also attenuated the Aβ-stimulated increases in active, phosphorylated Lyn and Src levels but not ERK in BV2 cells. Finally, LDDN-0003499 was able to dose-dependently attenuate Aβ stimulated TNF-α and IL-6 secretion.

## Methods

### Human Tissue

Use of human tissue was approved by the University of North Dakota Institutional Review Board (IRB-200412-198). A consent form was waived based upon the procurement from the University of Washington Alzheimer’s Disease Research Center tissue bank. The human tissue was obtained from the University of Washington Alzheimer Disease Research Center tissue bank (ADRC, NIH P50AG05136 (http://depts.washington.edu/adrcweb/).

### Antibodies and Reagents

The anti-Lyn, anti-Src, anti-α-tubulin antibodies and horseradish peroxidase conjugated secondary antibodies were purchased from Santa Cruz Biotechnology (Santa Cruz, CA). The anti-mouse TNF-α, IL-6, and IL-1β ELISA kits were obtained from R&D Systems (Minneapolis, MN). Anti-phospho-tyrosine (4G10) antibody was from Upstate (Temecula, CA), and anti-pLyn (Tyr 396) antibody was purchased from Abcam (Cambridge, MA). Anti-pSrc, Src, pERK, pJNK, p-p38, JNK, p38 and ERK2 antibodies were purchased from Cell Signaling Technology (Danvers, MA). LPS (Lipopolysaccharides from *E*.*Coli* 026:B6) was obtained from Sigma-Aldrich Corp. (St. Louis, MO). The LDH cytotoxicity assay kit was from Promega Corporation (Madison, WI). Human Aβ1–42 was purchased from rPeptide (Bogart, GA).

### Compound Library

The LDDN compound library had been used for a high-throughput screen assay to identify inhibitors of Lyn kinase activity. Results from this prior screen had identified four compounds with potential Lyn/Src family kinase inhibitory ability. The library consists of 150,000 compounds purchased from multiple commercial vendors as well as sets of proprietary compounds and has been designed with various computational filters to select compounds with an increased probability of oral bio-availability and blood brain barrier (BBB) penetration, which includes calculations of Polar Surface Area (physico-chemical descriptor that strongly correlates with oral bio-availability and the ability to cross the BBB), Lipinski’s “rule of five”, and other desirability filters. A subset of the LDDN chemical library consisting of about 75,000 compounds was used for this screen and consists of the Prestwick collection of FDA-approved drugs, an NINDS collection of known bioactive compounds, purified compounds from natural sources, peptides and small molecules purchased from Peakdale, Maybridge, Cerep, ChemBridge, Bionet, Prestwick, SPECS and Chemical Diversity Lab Inc. Furthermore, they were chosen for the following additional properties: low proportions of compounds that contain known toxicophores, low proportion of compounds containing reactive functional groups and maximization of molecular diversity. In addition, the LDDN has collected proprietary compounds from various academic labs throughout the world and provided some of their own unique compounds synthesized by LDDN chemists.

### Aβ Preparation

Each vial of Aβ1–42 (NaOH salt) obtained from rPeptide (Bogart, GA) was re-suspended in sterile water to a concentration of 250μM Aβ, aliquoted and stored at -20°C until use.

### BV2 Cell Line

Immortalized murine microglial BV2 cells, obtained from Dr. Gary E. Landreth, Cleveland, Ohio, were maintained in 100-mm dishes in DMEM/F12 (Gibco RBL, Rockville, MD) supplemented with 10% heat-inactivated fetal bovine serum (FBS) (U.S. Biotechnologies Inc., Parkerford, PA), 5% horse serum (Equitech-Bio, Inc., Kerrville, TX), penicillin G (100 units/ml), streptomycin (100 mg/ml), and L-glutamine (2 mM). These cells were originally transformed from murine microglia cultures using a v-raf/v-myc carrying retrovirus [44]. The cells were plated at 3 × 10^6^ cells/dish and incubated at 37°C in a humidified atmosphere containing 5% CO_2_ and 95% air.

### Cell Stimulation

BV2 cells were harvested and counted before stimulation. The cells were untreated (control), vehicle-treated (DMSO), or treated with increasing concentrations (0.5nM, 5nM, 50nM, 0.5 μM, 5μM and 50 μM) of LDDN-0003499, LDDN-0075935, LDDN-0125694 or LDDN-0127164 for 24h to assess toxicity and 1 hour to assess effects on basal kinase activity. For secretion analyses, cells were pretreated with drugs for 1 hour prior to stimulation with 50nM Aβ (overnight) or 25ng/mL LPS (overnight) in the presence of the drugs in serum free DMEM/F12. Media were collected for ELISA analysis and for LDH release viability assays. For signaling analyses, cells were pretreated with drugs for 1 hour prior to 5 minute stimulation with 1μM Aβ 1–42. Following the 5 minute stimulations, cells were lysed for western blot analysis using radioimmunoprecipitation assay buffer (RIPA) (20mM Tris, pH 7.4, 150mM NaCl, 1mM Na3VO4 10mM NaF, 1mM EDTA, 1mM EGTA, 0.2mM phenylmethylsulfonyl fluoride, 1% triton X-100, 0.1% SDS, and 0.5% deoxycholate) with protease inhibitors (AEBSF 104mM, Aprotinin 0.08mM, Leupeptin 2.1mM, Bestatin 3.6mM, Pepstatin A 1.5mM, E-64mM). Protein concentrations were determined using the Bradford method [45].

### Cell Viability Assays (LDH release assay)

For the lactate dehydrogenase release (LDH) assay, media following the 24 hour cell stimulations were transferred onto a new 96-well plate and LDH concentrations assayed according to manufacturer instructions (Promega Corporation, Madison, WI). Background absorbance was subtracted from each condition. All absorbance values for the LDH assay were averaged and plotted ±SD.

### Enzyme-Linked Immunosorbent Assay (ELISA)

Media were collected from BV2 cells following 24 hour stimulations for ELISA analyses. Levels of mouse TNF-α, IL-6, and IL-1β in the media were determined using commercially available ELISA kits according to the manufacturer’s protocol (R&D Systems).

### Western Blot

Cell lysates were resolved by SDS-PAGE on 10% polyacrylamide gels and transferred to PVDF membranes. Western blots were blocked with 3% bovine serum albumin and incubated in anti-phospho-tyrosine (4G10), anti-pSrc (Tyr 416), anti-pERK, anti-pLyn (Tyr 396), anti-pJNK, and anti-phospho-p38 primary antibodies with anti-α-tubulin, Src, ERK2, Lyn, JNK, and p38 antibodies used as the respective loading controls. Blots were washed followed by incubation with HRP-conjugated secondary antibodies and antibody binding was detected via enhanced chemiluminescence (GE Healthcare, Piscataway, NJ). Western blots were quantified using Adobe Photoshop software. Optical densities of bands were normalized using their appropriate loading controls and averaged (±) SD.

### Tissue Immunostaining

Human tissue (3 AD and 3 age-matched control brains) was obtained from University of Washington Alzheimer Disease Research Center (ADRC, NIH P50AG05136) and was cut via freezing microtome into 40μm sections for immunostaining. Sections of AD brain tissue along with age-matched control were immunostained against anti-p-Lyn antibody using Vector SG (Grey) chromogen (Vector Laboratories) as the first chromogen. The sections were then stripped using 0.2 N HCl, 5 minutes and incubated with the second primary antibody (anti-Aβ, 4G8) for immunostaining using Vector Red (Vector Laboratories) as the second chromogen.

### *In Vitro* ADME

In order to help translate the *in vitro* analyses of LDDN-0003499 for future *in vivo* study, *in vitro* ADME (Absorption, Distribution, Metabolism and Excretion) analyses for LDDN-0003499 were performed by Cyprotex US, LLC (Watertown, MA). Microsomal stability for the compound was first determined using both human and mouse liver microsomes via a microsomal stability assay in the presence or absence of the cofactor NADPH (and compared to a high clearance control, verapamil, and a low clearance control, warfarin). Briefly, the compound was incubated with human or mouse microsomes in the presence or absence of NADPH. The aliquots of this reaction mixture were collected at different time intervals and subjected to LC-MS/MS analysis to quantitate the amount of the remaining parent LDDN-0003499 compound. Data were converted to percent remaining by dividing by the time zero concentration value. *In vitro* half-life was determined based on the slope of the (first-order) decay curve and this value was converted to intrinsic clearance (CLint). In addition, to determine likely oral absorption of LDDN-0003499 and to predict involvement of the P-gp efflux transporter (a well-known barrier to blood brain barrier (BBB) permeability) the compound was tested in a Caco-2 bidirectional permeability assay. Briefly, Caco-2 cells were plated in Millipore 96-well transwell plates and allowed to differentiate for 21 days to form polarized, epithelial monolayers. For apical to basolateral (A→B) permeability, the compound was added to the apical (A) side and amount permeation determined on the basolateral (B) side. For B→A permeability, the compound was added on the basolateral (B) side. Upon completion of the incubation (2 hr), the receiver side buffer was removed for analysis by LC-MS/MS. From the samples, apparent permeability rate coefficients (Papp values) and efflux ratios were determined for the compound.

### Statistical Analysis

Data are presented as mean ± standard deviation. Values statistically different from controls were determined using one-way ANOVA. The Turkey-Kramer multiple comparisons post-test was used to determine p values.

## Results

### Effects of the LDDN compounds on cell viability

**Fig 1:**
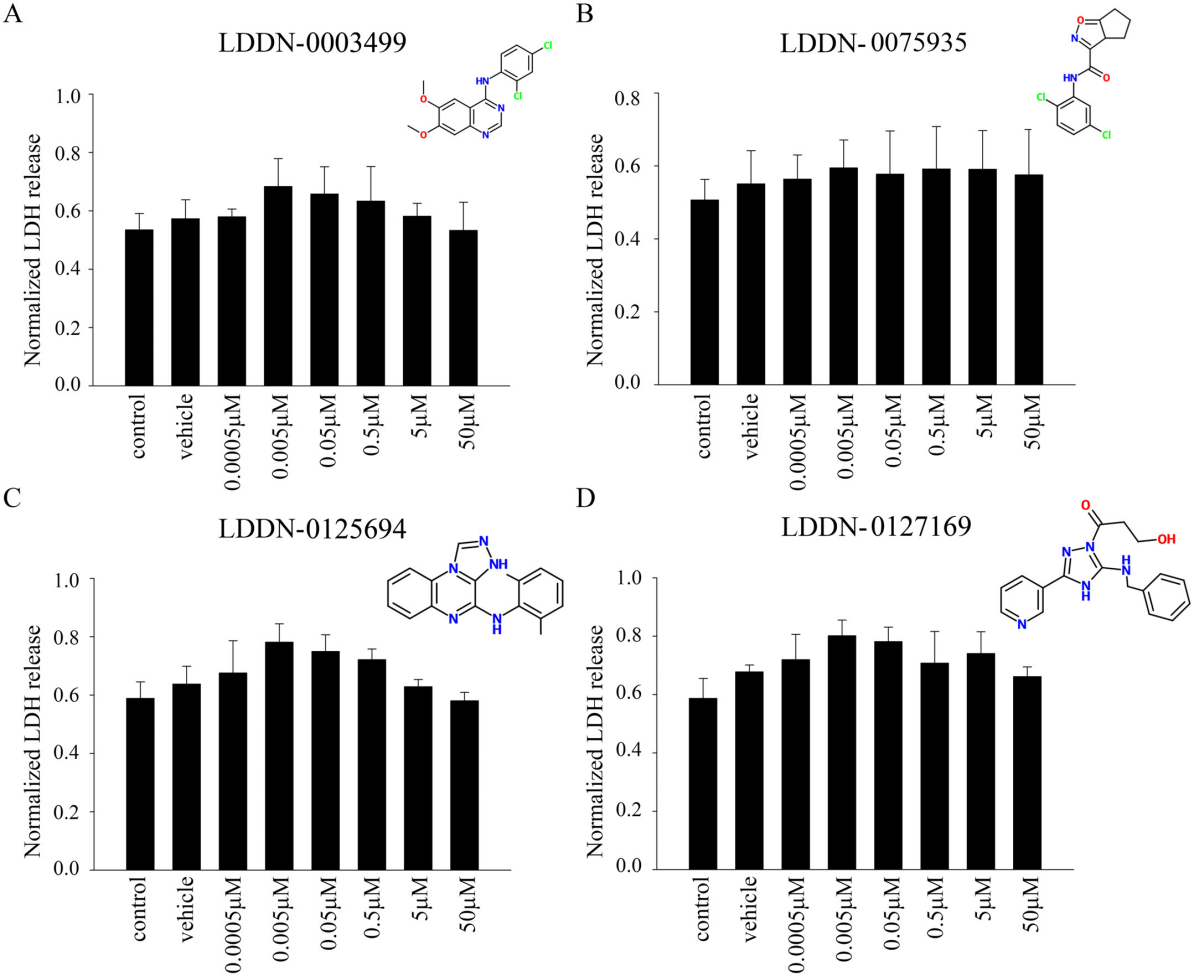
LDDN compounds were not toxic to microglial BV2 cells. Microglial BV2 cells were untreated (control), vehicle DMSO treated, or treated with 0.5nM, 5nM, 50nM, 0.5 μM, 5μM and 50 μM (A) LDDN-0003499, (B) LDDN-0075935, (C) LDDN-0125694, and (D) LDDN-0127164 for 24h. Following compound treatment, media were used to perform LDH release assay to determine cell viability. Three independent experiments with 8 replicates each were performed and absorbance values graphed and averaged ± SD.

Although all four of the LDDN compounds had been characterized as possible Lyn inhibitory molecules from prior cell-free activity assays, their efficacy in cells had not yet been assessed. Therefore, before quantifying their ability to alter cellular kinase activities, we first determined whether the compounds exhibited any microglial toxicity. To perform this assessment we elected to use the common microglial cell line model, BV2 cells. They were treated with increasing concentrations of LDDN-0003499, LDDN-0075935, LDDN-0125694 and LDDN-0127164 from 0.5 nM up to 50 μM for 24h. The media were removed and used for LDH release assays to determine cell viability. Quantitation of LDH released from the media following compound treatment demonstrated that none of the compounds were toxic at any of the concentrations tested (Fig 1). For subsequent experiments assessing effects on tyrosine kinase inhibition, the concentration range of 0.5nM, 5nM, 50nM, 0.5μM, 5μM and 50μM was chosen for treatment of the BV2 cells.

### LDDN-0003499 treatment decreased total protein phosphotyrosine levels in microglia BV2 cells

**Fig 2:**
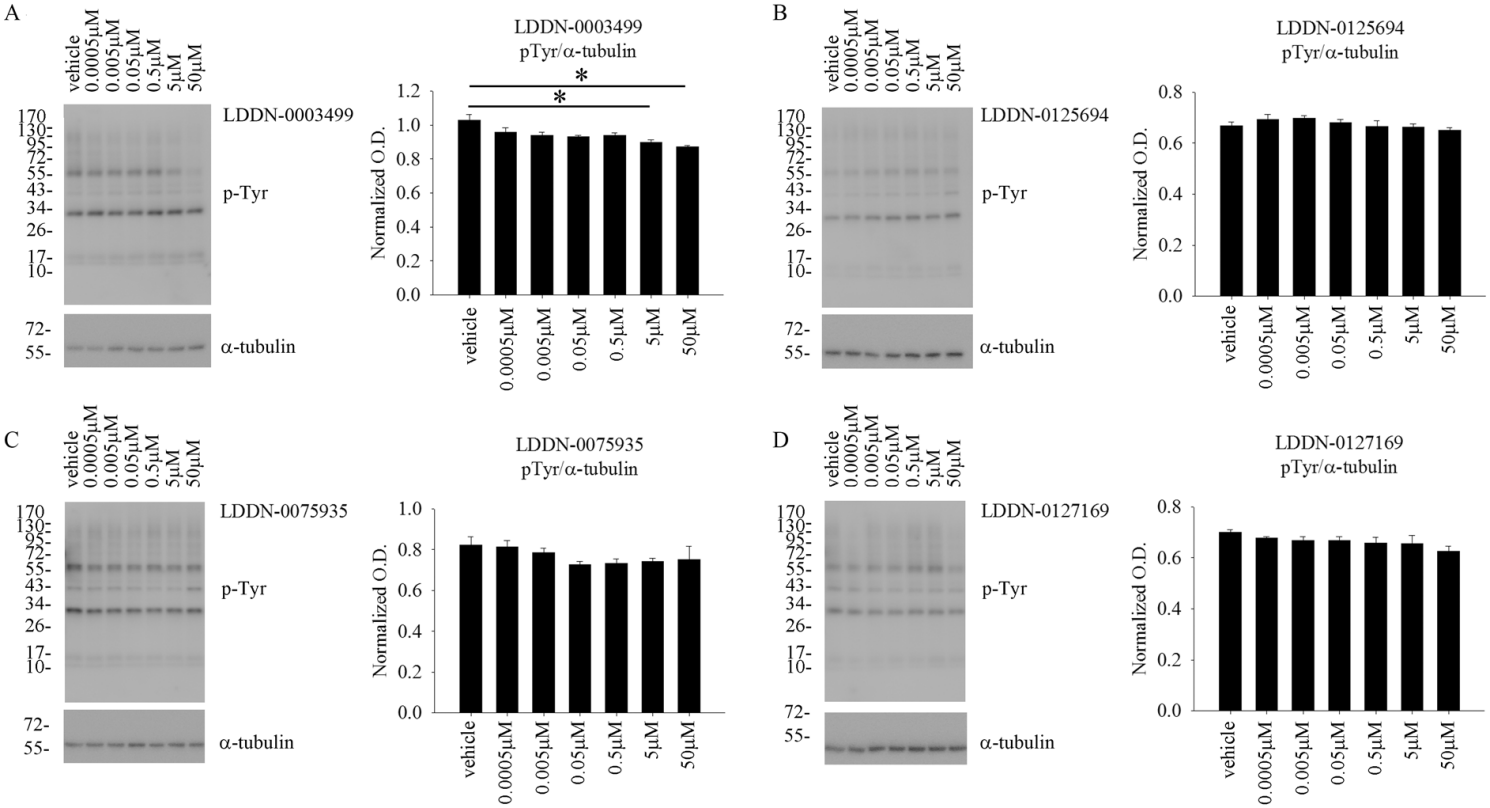
LDDN-0003499 treatment attenuated total protein phosphotyrosine levels in BV2 cells. Microglial BV2 cells were treated with vehicle (DMSO), 0.5nM, 5nM, 50nM, 0.5 μM, 5μM, and 50 μM (A) LDDN-0003499, (B)LDDN-0125694, (C) LDDN-0075935, and (D) LDDN-127164 for 1h. Cell lysates were resolved via SDS-PAGE and western blotted using anti-phosphotyrosine (4G10) antibody with α-tubulin as the loading control. A representative western blot is shown. Optical densities from three independent experiments were graphed and averaged ± SD (*p<0.05 vs. vehicle).

In order to validate a tyrosine kinase inhibitory ability of the compounds in cells, the BV2 cells were again treated with increasing concentrations of LDDN-0003499, LDDN-0075935, LDDN-0125694 and LDDN-0127164. Of the four compounds analyzed using western blot analyses, LDDN-0003499 was able to significantly decrease the total protein phosphotyrosine levels in a dose-dependent manner as compared to vehicle treated cells (Fig 2). LDDN-0125694, LDDN-0075935, and LDDN-0127164 treatments had no effect on protein phosphotyrosine levels at the concentrations used (Fig 2).

### LDDN-0003499 treatment dose-dependently attenuated protein levels of active, phosphorylated Lyn and Src in BV2 cells

**Fig 3:**
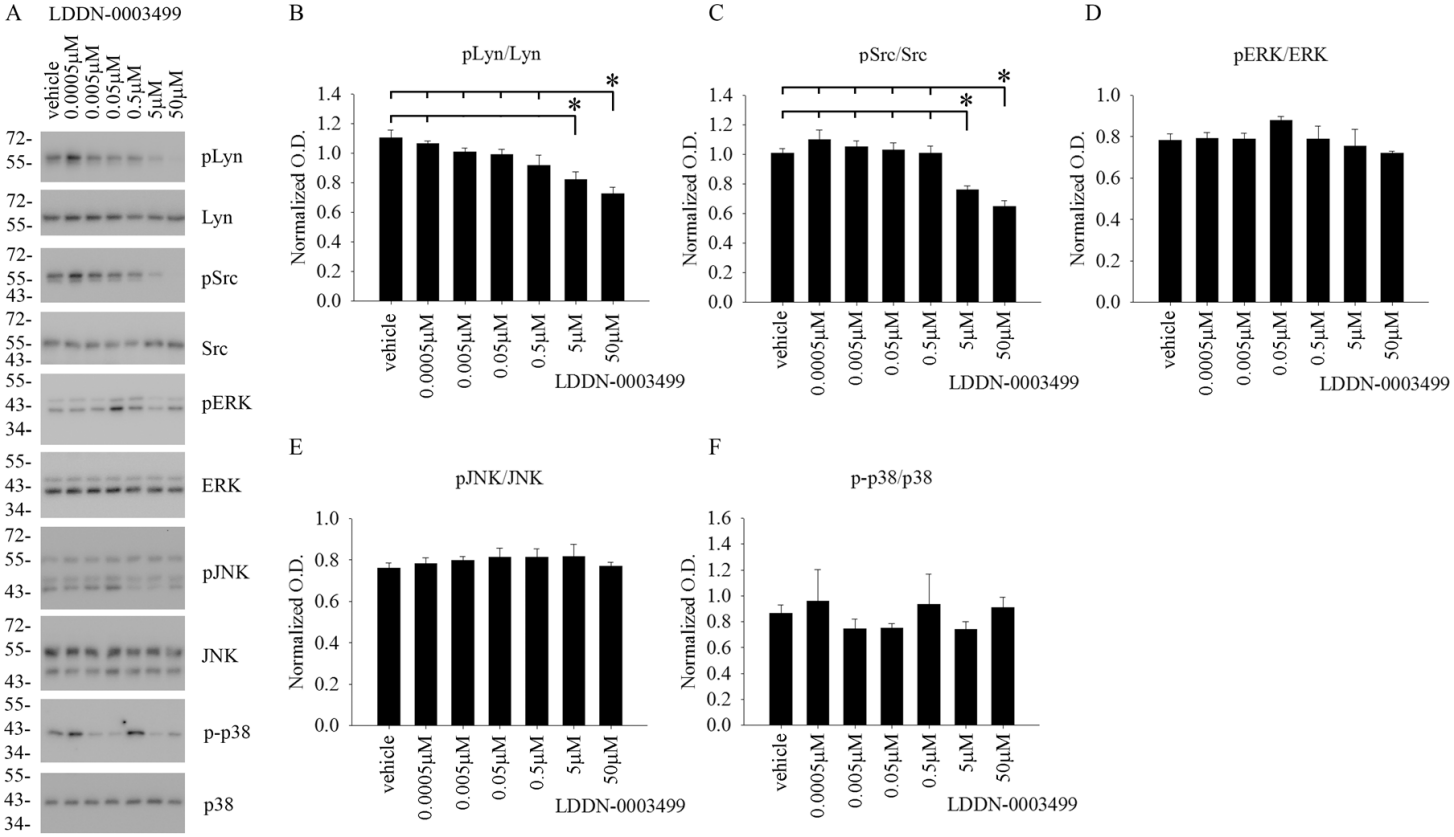
LDDN-0003499 treatment attenuated active, phosphorylated Lyn and Src but not ERK, JNK, or p38 protein levels in BV2 cells. Microglial BV2 cells were treated with vehicle (DMSO), 0.5nM, 5nM, 50nM, 0.5 μM, 5μM, and 50 μM LDDN-0003499 for 1h. Cells lysates were used for western-blot analyses with (B) anti-pLyn (Tyr 396), (C) anti-pSrc (Tyr 416), (D) anti-pERK, (E) anti-pJNK, and (F) anti-p-p38 antibodies with Lyn, Src, ERK2, JNK, and p38 antibodies as their respective loading controls. Optical densities from three independent experiments were graphed and averaged ± SD (*p<0.05 vs. vehicle). (A) A representative western blot is shown.

**Fig 4:**
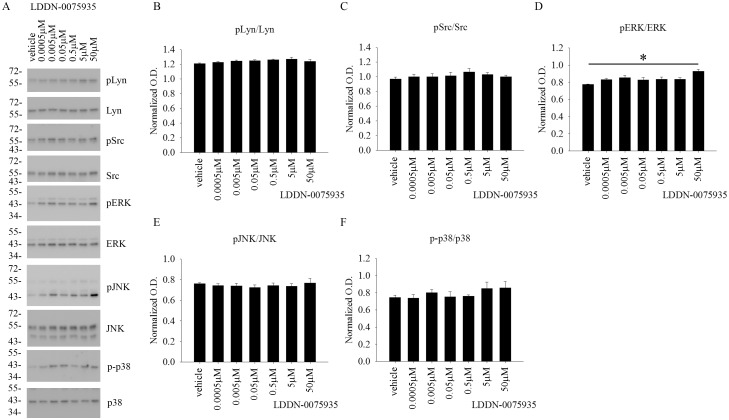
LDDN-0075935 treatment did not attenuate active, phosphorylated Src, Lyn, ERK, JNK, or p38 protein levels in BV2 cells. Microglial BV2 cells were treated with vehicle (DMSO), 0.5nM, 5nM, 50nM, 0.5 μM, 5μM, and 50 μM LDDN-0075935 for 1h. Cells lysates were used for western-blot analyses with (B) anti-pLyn (Tyr 396), (C) anti-pSrc (Tyr 416), (D) anti-pERK, (E) anti-pJNK, and (F) anti-p-p38 antibodies with Lyn, Src, ERK2, JNK, and p38 antibodies as their respective loading controls. Optical densities from three independent experiments were graphed and averaged ± SD (*p<0.05vs. vehicle). (A) A representative western blot is shown.

**Fig 5:**
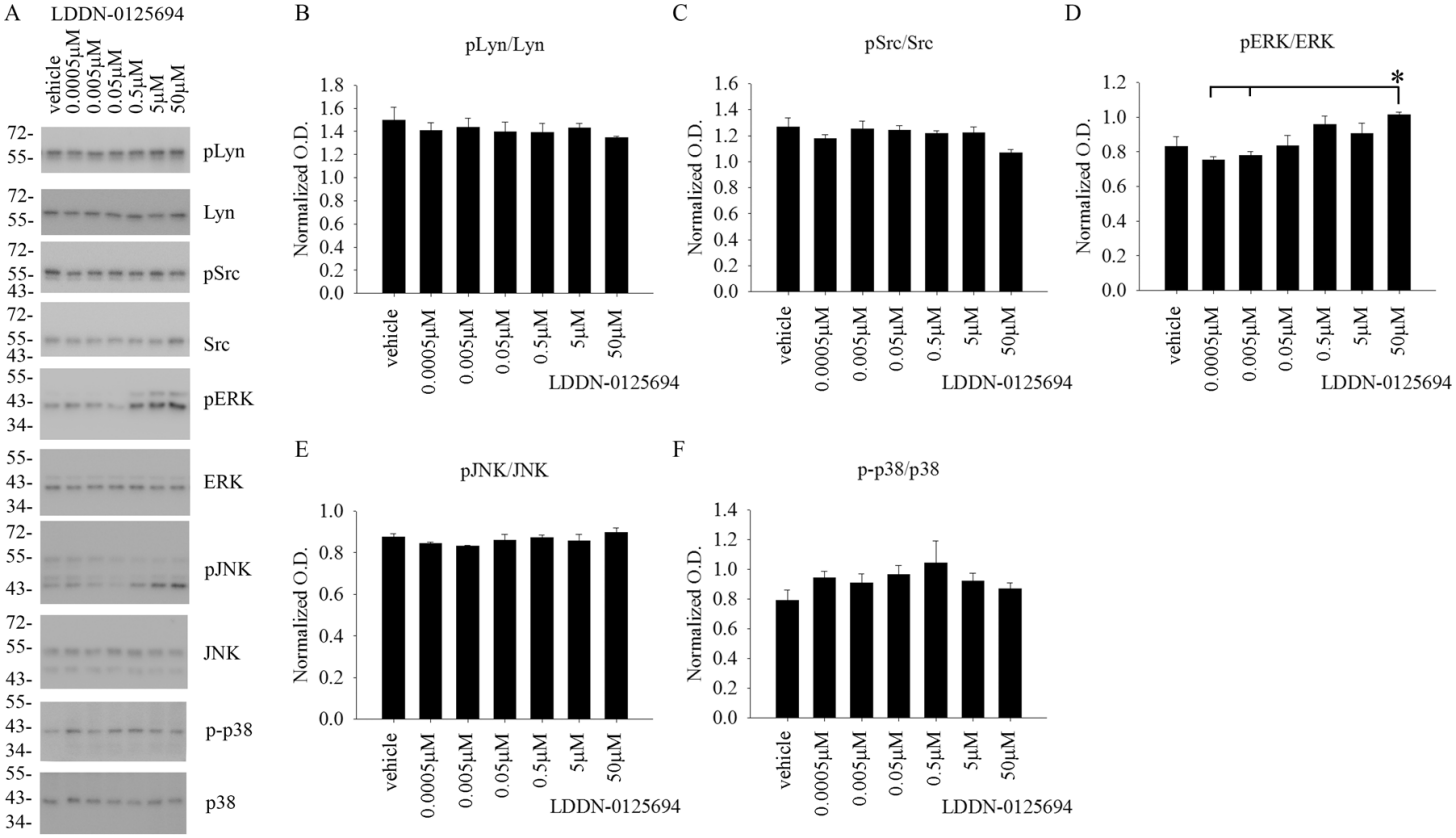
LDDN-0125694 treatment did not attenuate active, phosphorylated Src, Lyn, ERK, JNK, or p38 protein levels in BV2 cells. Microglial BV2 cells were treated with vehicle (DMSO), 0.5nM, 5nM, 50nM, 0.5 μM, 5μM, and 50 μM LDDN-0075935 for 1h. Cells lysates were used for western-blot analyses with (B) anti-pLyn (Tyr 396), (C) anti-pSrc (Tyr 416), (D) anti-pERK, (E) anti-pJNK, and (F) anti-p-p38 antibodies with Lyn, Src, ERK2, JNK, and p38 antibodies as their respective loading controls. Optical densities from three independent experiments were graphed and averaged ± SD (*p<0.05 vs. vehicle). (A) A representative western blot is shown.

**Fig 6:**
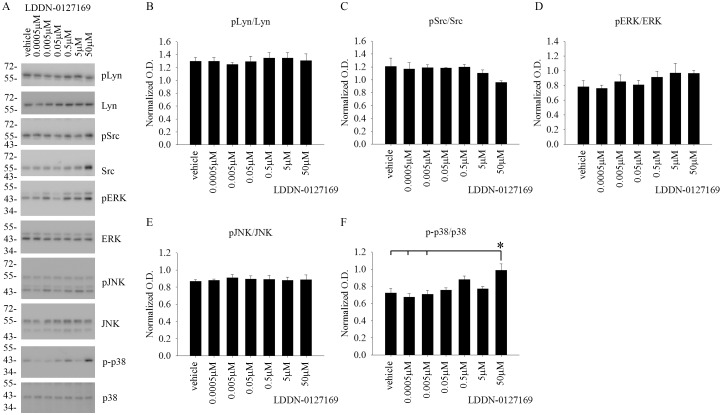
LDDN-0127164 treatment did not attenuate active, phosphorylated Src, Lyn ERK, JNK, or p38 protein levels in BV2 cells. Microglial BV2 cells were treated with vehicle (DMSO), 0.5nM, 5nM, 50nM, 0.5 μM, 5μM, and 50 μM LDDN-0075935 for 1h. Cells lysates were used for western-blot analyses with (B) anti-pLyn (Tyr 396), (C) anti-pSrc (Tyr 416), (D) anti-pERK, (E) anti-pJNK, and (F) anti-p-p38 antibodies with Lyn, Src, ERK2, JNK, and p38 antibodies as their respective loading controls. Optical densities were graphed and averaged ± SD (*p<0.05 vs. vehicle). (A) A representative western blot is shown.

Although the compounds were tested for an ability to decrease total protein phosphotyrosine levels, the *in vitro* cell-free kinase assay target for these compounds was the Src family of kinases. Therefore, in order to identify any ability to inhibit the Src family of kinases within cells, BV2 cells were again treated with increasing concentrations of LDDN-0003499, LDDN-0075935, LDDN-0125694 and LDDN-0127164 for 1h. Cell lysates were analyzed using western blots to quantify changes in pLyn, pSrc, pERK, pJNK, and p-p38 levels. Quantitation of the blots demonstrated a dose-dependent decrease in active, phosphorylated Lyn (pLyn) and Src (pSrc) protein levels in the LDDN-0003499 treated cells (Fig 3). LDDN-0075935, LDDN-0125169 or LDDN-0127164 treatments were not able to attenuate pLyn or pSrc protein levels at any of the concentrations tested (Figs 4–6). As unrelated kinase controls, each compound was tested for any ability to alter active levels of MAP kinases extracellular signal regulated kinase 2 (ERK2), c-Jun N-terminal kinase (JNK), and p38 MAP kinase (p38). LDDN-0003499 had no ability to alter phosphorylated levels of ERK, JNK, or p38 (Fig 3). However, both LDDN-0075935 and LDDN-0125694 stimulated increased pERK protein levels at the highest drug concentration (Figs 4 and 5) while LDDN-0127169 stimulated increased phosphorylated p38 protein levels at the highest drug concentration (Fig 6). These data suggested that LDDN-0003499 may be a promising compound for selectively inhibiting the Src family of kinases in microglia. To assess the ability of LDDN-0003499 to attenuate stimulated rather than basal kinase activation, BV2 cells with stimulated with Aβ or LPS and increasing concentrations of LDDN-0003499 in the subsequent experiments.

### LDDN-0003499 treatment attenuated the Aβ-dependent increase in protein levels of active phosphorylated Src and Lyn but not pERK in BV2 cells

**Fig 7:**
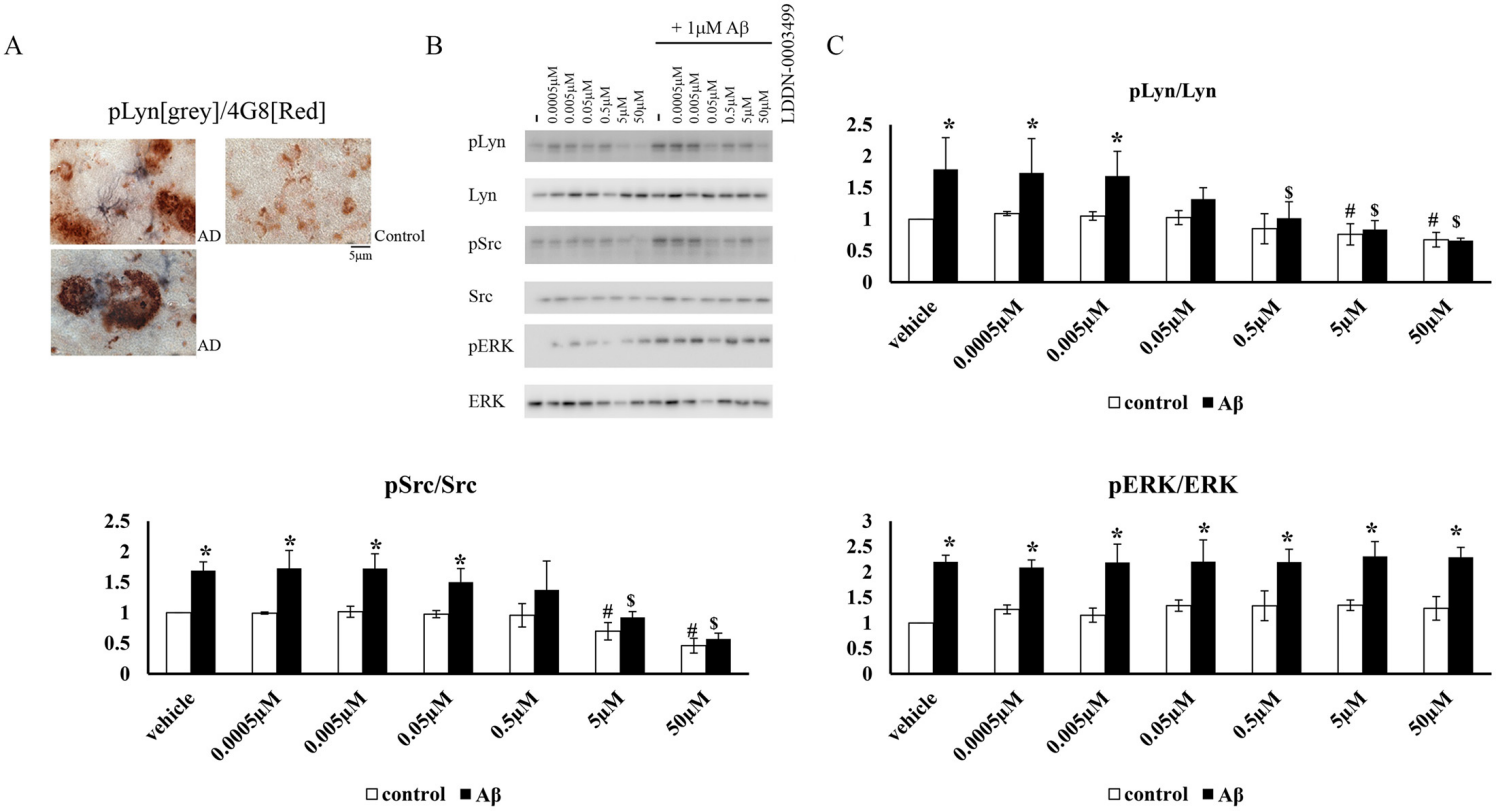
LDDN-0003499 treatment attenuated an Aβ-dependent increase in active phosphorylated Src and Lyn but not pERK protein levels in BV2 cells. (A) Human AD temporal lobe sections along with age-matched controls were immunostained using anti-pLyn antibody and anti-Aβ (4G8) antibody. A double label with anti-pLyn (grey) and anti-Aβ (red) antibodies is shown. (B) BV2 cells were treated with vehicle DMSO (v) or 1 μM Aβ in presence and absence of increasing concentrations of LDDN-0003499 (0.0005, 0.005, 0.05, 0.5, 5, 50 μM). Cell lysates were resolved by SDS-PAGE and western blotted using anti-pSrc, anti-pLyn and p-ERK antibodies with Src, Lyn, and ERK as loading controls. (C) Optical densities from three independent experiments were averaged and graphed ± SD (*p<0.05 respective control vs Aβ, $p<0.05 vs. Aβ only, #p<0.05 vs. vehicle).

As already mentioned, a primary rationale for this study effort was to identify a small molecule capable of decreasing Aβ-mediated stimulation of microglia. Active levels of both Lyn and Src increase *in vitro* upon Aβ stimulation of microglia and monocytes [24, 39, 40] and Aβ plaque associated microglia have increased phosphotyrosine immunoreactivity in AD human brains [40] and its mouse models [39]. Moreover, we previously demonstrated that plaque associated microglia have elevated levels of pSrc in human diseased brains [40]. We have found a similar increase in immunoreactivity of phosphorylated, active Lyn in plaque associated microglia in human AD brains (Fig 7). Therefore, we expected LDDN-0003499 to be able to attenuate Aβ-stimulated microglial activation in correlation with its ability to inhibit Src/Lyn activation. To test this idea, BV2 cells were stimulated with Aβ in the absence or presence of increasing concentrations of LDDN-0003499. As expected, Aβ stimulation significantly increased pLyn, pSrc, and pERK protein levels. Pretreatment with LDDN-0003499 attenuated protein levels of pLyn and pSrc basally and with Aβ stimulation (Fig 7). Most importantly, 50nM LDDN-0003499 was sufficient to significantly attenuate the Aβ stimulated increase in pLyn levels while 500nM was required to attenuate pSrc. This demonstrated tenfold selectivity for Lyn versus Src in the Aβ stimulation paradigm. However, consistent with the inability of the drug to attenuate phosphorylated MAP kinases levels (Fig 3), examining pERK levels as a representative MAP kinase showed no inhibition following Aβ stimulation (Fig 7).

### LDDN-0003499 treatment attenuated the Aβ-dependent increase in secretion of the pro-inflammatory cytokines, TNF-α and IL-6

**Fig 8:**
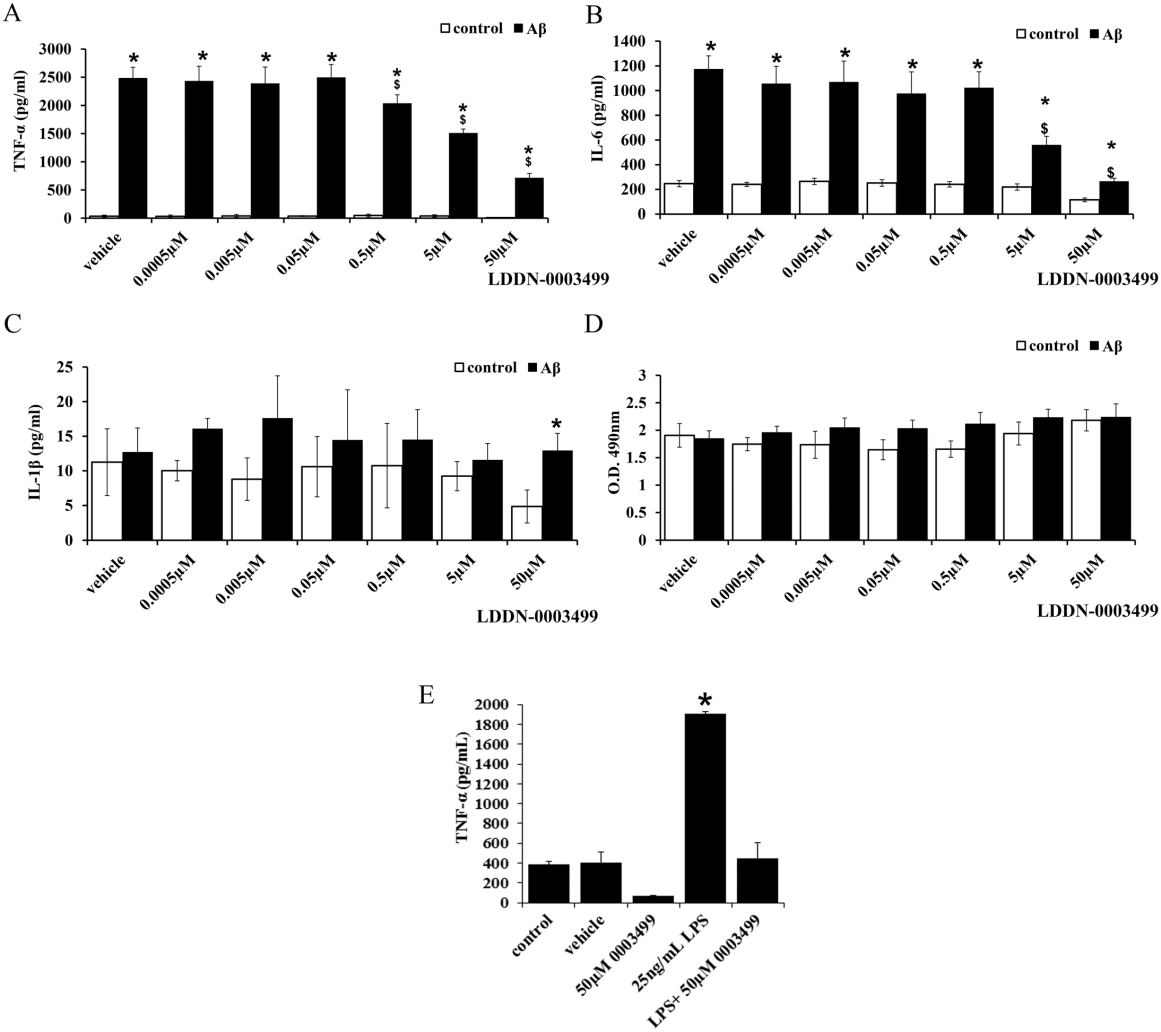
LDDN-0003499 treatment attenuated Aβ-dependent microglial TNF-α and IL-6 secretion. Microglial BV2 cells were pretreated for 1 hour with DMSO vehicle (v) or varying concentrations of LDDN-0003499 then stimulated with (A, B, C) 50nM Aβ 1–42 or (E) 25ng/ml LPS in the maintained presence/absence of LDDN-0003499. Media were collected from the cells following 24h stimulation and used to quantify changes in TNF-α (A, E), IL-6 (B) or IL-1β (C) secretion via ELISA. Secreted values from three independent experiments were averaged ± SD (*p< 0.05 respective control vs Aβ, $p<0.05 vs. Aβ only). (D) Media from treated cells were also used assess cell viability via the LDH release assay. Absorbance values were averaged ±SD.

Prior work from our group [39] and others [34, 46, 47] has demonstrated that Aβ stimulates microglia cytokine secretion via a Src family kinase-dependent pathway. In order to determine whether LDDN-0003499 was able to inhibit the Aβ-stimulated acquisition of a reactive secretory, phenotype microglia were treated with Aβ for 24 h in the absence or presence of LDDN-0003499. As predicted, Aβ stimulated a significant increase in TNF-α (Fig 8) and IL-6 (Fig 8) secretion that was dose-dependently inhibited by treatment with LDDN-0003499 beginning with 500nM for TNF-α. Surprisingly, Aβ stimulated no increase in IL-1β secretion by the BV2 cells perhaps reflecting a difference between the cell line and primary microglia (Fig 8). LDDN-0003499 treatment also attenuated 24h LPS stimulated TNF-αsecretion by microglia confirming that the anti-inflammatory effect of the compound was not stimulus specific (Fig 8).

### LDDN-0003499 has the properties suitable for a drug to be orally available and BBB penetrant

Based upon the favorable anti-inflammatory properties of LDDN-0003499 we next began evaluating its potential for future *in vivo* use. In order to begin predicting *in vivo* properties of LDDN-0003499, it was tested in a microsomal stability assay (Table 1) and a Caco-2 permeability assay (Table 2). Both human and mouse liver microsomal stability of compound LDDN-0003499 was quantified in the presence or absence of NADPH. Since the liver is the primary site of drug metabolism, subcellular fractions of liver microsomes are useful *in vitro* models of hepatic clearance as they still contain many of the drug metabolizing enzymes found in the liver (notably, the cytochrome P450 enzymes). Highly cleared compounds *in vitro* are likely to be rapidly cleared *in vivo* resulting in a short duration of action. Although the compound was relatively resistant to non-NADPH dependent enzymatic clearance (half-life of 65 and 88 min. for human versus mouse microsomes, respectively) the compound had moderately high NADPH dependent clearance in both human and mouse microsomes with a half-life of 14.1 and 16.6 min. for human versus mouse, respectively (Table 1). However, a Caco-2 permeability assay demonstrated a high apparent permeability rate coefficient (Papp) of 19.0 (A→B) and 16.2 (B→A) suggesting that LDDN-0003499 may be orally absorbed in both mouse model and human studies (Table 2). An efflux ratio (RE) of >2 indicates that a compound may be a potential substrate for permeability glycoprotein (P-gp) or other active transporters. P-gp is an ATP-dependent efflux pump capable of transporting a wide range of substrates allowing it to transport drugs back into the intestinal lumen or back into brain capillaries, for example. The low efflux ratio of 0.85 for LDDN-0003499 suggests that this compound is not a potential substrate for P-gp and therefore has improved potential for oral absorption and BBB permeation. Although clearance of the compound was moderately high, the high permeability and low efflux properties are encouraging for future *in vivo* studies.

**Table 1:** Microsomal Intrinsic Clearance. LDDN-0003499 was assessed for human and mouse microsomal clearance using a microsomal intrinsic clearance assay.

Test Article	Test Conc.	Species	NADPH-Dependent CL_int_ (μl min^-1^ mg^-1^)	NADPH-Depend. T_1/2_ (min)	NADPH-Free CL_int_ (μl min^-1^ mg^-1^)	NADPH-Free T_1/2_ (min)	Comment
**LDDN-0003499**	1 μM	Human	98.4	14.1	21.2	65	
	1 μM	Mouse	83.3	16.6	15.8	88	
verapamil	1 μM	Human	120	11.6	18.1	77	high clearance control
	1 μM	Mouse	297	4.7	<5.8	>240	
warfarin	1 μM	Human	9.6	145	11.5	121	low clearance control
	1 μM	Mouse	10.3	134	8.1	170	

CL_int_: Microsomal Intrinsic Clearance. T_1/2_: Half-Life.

**Table 2:** Caco-2 Permeability. LDDN-0003499 was assessed for human oral availability and blood brain barrier penetration using a Caco-2 permeability assay.

Test Article	Test Conc.	Assay Duration	mean A→B P_app_ (10^-6^ cm s^-1^)	mean B→A P_app_ (10^-6^ cm s^-1^)	Efflux Ratio	Comment
ranitidine	10 μM	2 hr	0.30	3.1	10.3	low permeability control
warfarin	10 μM	2 hr	27.1	10.4	0.38	high permeability control
talinolol	10 μM	2 hr	0.071	9.8	138	P-gp efflux control
**LDDN-0003499**	10 μM	2 hr	19.0	16.2	0.85	**high permeability**

Papp: apparent permeability rate coefficient. Efflux Ratio: P_app_(B→A) / P_app_(A→B).

## Discussion

These data extend our previous efforts to identify small molecules able to inhibit particularly Src family tyrosine kinase activities following microglia-Aβ interaction for the purpose of anti-inflammatory therapeutic discovery [39]. Our prior work has demonstrated increased non-receptor tyrosine kinase activation including Src, Lyn and Syk kinases during Aβ-mediated microglia stimulation in cultures and *in vivo* using an APP/PS1 mouse model of AD [36, 39, 40]. A number of non-receptor tyrosine kinase inhibitors have been demonstrated to inhibit Aβ-stimulated tyrosine kinase mediated microglial activation including PP1 [34, 48], piceatannol [24, 34, 49, 50], and dasatinib [39, 40] to list only a few. In this study we sought to determine whether additional molecules can be identified with somewhat higher selectivity for Lyn versus other non-receptor tyrosine kinases in the Src family due to fact that Lyn is immune cell enriched [41–43] and less off target effects might be expected with a molecule selectively inhibiting Lyn versus other family members. In our prior work we found that a clinically available broad Src family kinase inhibitor, dasatinib, was sufficient to inhibit microgliosis both *in vitro* and *in vivo* in response to Aβ stimulation [39]. However, dasatinib can inhibit numerous non-receptor tyrosine kinases including Src, Lyn, Fyn, Yes, and Bcr/Abl raising the possibility that long-term treatment, as might be required for treating AD patients, would suffer from adverse consequences [51, 52].

In order to begin the process of identifying a molecule that might demonstrate selectivity for one Src family member versus another, a high throughput Lyn kinase activity assay had been previously performed screening 75,000 compounds. This cell-free screening identified four inhibitory compounds that were tested in the current cell-based study. Of the four compounds, LDDN-0003499 inhibited active, phosphorylated Lyn and Src kinase levels in non-stimulated cells suggesting no basal specificity for a particular Src family member. In addition, the compound had no effect on basal phosphorylated levels of ERK, JNK, or p38 suggesting some specificity for tyrosine kinases. The compound also inhibited Aβ-stimulated increases in both pSrc and pLyn but not pERK levels indicating that it attenuated Src but not MAP kinase family activation. Importantly, it had some selectivity for inhibiting Lyn versus Src following Aβ stimulation differing from its effects on basal kinase activity. It also significantly inhibited TNF-α and IL-6 secretion in response to Aβ stimulation as well as LPS-stimulated TNFα secretion verifying potent anti-inflammatory actions on microglia that were not limited to simply Aβ stimulation.

In spite of our original focus on Lyn activity as an attractive regulator of Aβ-stimulated microgliosis due to its high level of expression in immune cells, we are aware that numerous additional kinases including Src are involved in the stimulated increase in cytokine secretion. In addition, it is not yet clear whether inhibition of any particular Src family member is therapeutically more attractive than another. The compound was somewhat selective for Lyn over Src and was apparently selective for inhibiting tyrosine kinases compared to MAP kinases. This suggests some specificity of action and implies that Aβ-stimulated MAP kinase activation is not entirely dependent upon membrane proximal Src kinase activation. Future broad screening studies to quantify the inhibitory ability of LDDN-03499 for both tyrosine and serine/threonine kinases will better elucidate its mechanism of action in the cells. The present study was aimed at characterizing the drugs in an *in vitro* system but we appreciate that further structural modifications may be required to improve the characteristics of LDDN-0003499 before *in vivo* studies can be performed. Nevertheless, our preliminary *in vitro* ADME data suggest that the compound in its current form will be bioavailable and brain penetrant. The main chemical backbone of LDDN-0003499 is 4-anilinoquinazoline (Fig 1) which, when substituted with different moieties, has been used for numerous biological applications suggesting the future optimization may be required for *in vivo* testing of LDDN-0003499 [53, 54]. Needless to say, targeting Lyn and Src kinases for attenuating microglial activation during AD presents a promising opportunity for further drug development.

## Conclusions

This study characterized the ability of four different small molecules to inhibit Src family kinases in microglia basally and in response to Aβ stimulation. Based on the *in vitro* studies performed, we identified one compound, LDDN-0003499, with an ability to decrease basal pLyn and pSrc but not pERK, p-p38 and pJNK protein levels in BV2 cells. In addition, this compound attenuated Aβ-stimulated increases in both pSrc and pLyn in microglia correlating with robust inhibition of TNFα and IL-6 secretion. These data indicate that LDDN-0003499 is a Src family kinase inhibitor with potent anti-inflammatory effects. Further characterization of the compound both *in vitro* and *in vivo* as well as structural modification may yield a molecule of therapeutic interest.
